# Low reliability of the Risser sign in consecutive radiographs: a case series

**DOI:** 10.1186/1748-7161-8-S2-O18

**Published:** 2013-09-18

**Authors:** Stefano Negrini, Salvatore Atanasio, Sabrina Donzelli, Monia Lusini, Salvatore Minnella, Francesco Negrini, Fabio Zaina

**Affiliations:** 1 University of Brescia - IRCCS Don Gnocchi, Milan, Italy

## Background

The low reliability of the Risser sign has been described in previous studies; however, the test/retest reliability of the Risser sign in different radiographs has not been examined or reported.

## Purpose

The goal of this report was to present data collected from studying a group of adolescent idiopathic scoliosis (AIS) patients whose Risser sign decreased in two consecutive X rays.

## Methods

Case series using the European Risser test.

## Results

By chance, a reduction of the Risser test was discovered in one patient. Subsequently, other cases have been searched to see if this was an exceptional situation. In one year, we found three more cases.

**Table 1:** 

	1	2	3	4
Age	16.1	14.8	14.4	15.9
Height (cm)	0	+ 1	+1.5	0
Weight (kg)	+1	0	+1.5	0
Risser	4 to 2	1 to 0	2 to 1	4 to 3
Brace	Sforzesco	PASB	SpineCor	Sforzesco
Hours/day	23	12	20	16
Bracing months	4	6	4	22
Months between x-rays	7	6	6	14
In brace x-ray	No	No	Yes	No
Change of x-ray machine	No	No	Yes	Yes
°Cobb	-23	-4	-7	+4
Change of rotation of the pelvis	++	+	?	+
C7+L3 distance (cm)	-3	0	+0.5	-0.5
ATR (°)	-8	-3	-3	+3

## Conclusions and discussion

At this stage, the following explanatory hypotheses can be drawn:

Technical radiological differences (exposure, machine)

Variation of pelvis positioning

Postural changes influencing the pelvis

Brace compression on the pelvis

According to our study results, all of the hypotheses include data both in favor and against the reliability of the Risser sign. Since the Risser sign is a 2D evaluation of a 3D phenomenon, pelvis repositioning could perhaps be the most plausible explanation.

This case series is open to the possibility that the Risser sign is even less reliable than originally considered. Unfortunately, this result cannot be checked experimentally due to ethical reasons. Nevertheless, observational designs could be considered in the future.
